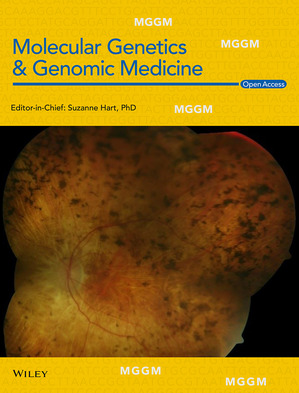# Cover

**DOI:** 10.1002/mgg3.2450

**Published:** 2024-05-08

**Authors:** Ryan J. German, Blake Vuocolo, Liesbeth Vossaert, Nichole Owen, Richard A. Lewis, Lisa Saba, The Texome Project, Michael F. Wangler, Sandesh Nagamani

## Abstract

The cover image is based on the Clinical Report *Novel hemizygous single‐nucleotide duplication in *RPGR* in a patient with retinal dystrophy and sensorineural hearing loss* by Ryan J. German et al., https://doi.org/10.1002/mgg3.2404